# Diazepam inhibits LPS-induced pyroptosis and inflammation and alleviates pulmonary fibrosis in mice by regulating the let-7a-5p/MYD88 axis

**DOI:** 10.1371/journal.pone.0305409

**Published:** 2024-06-14

**Authors:** Duanyi Song, Xuefang Tang, Juan Du, Kang Tao, Yun Li

**Affiliations:** Department of Anesthesiology, The Second People’s Hospital of Yunnan Province, Kunming, Yunnan, China; Universidade de Trás-os-Montes e Alto Douro: Universidade de Tras-os-Montes e Alto Douro, PORTUGAL

## Abstract

**Background and objective:**

Pulmonary fibrosis caused by lung injury is accompanied by varying degrees of inflammation, and diazepam can reduce the levels of inflammatory factors. Therefore, the purpose of this study was to determine whether diazepam can inhibit inflammation and ameliorate pulmonary fibrosis by regulating the let-7a-5p/myeloid differentiation factor 88 (MYD88) axis.

**Methods:**

Lipopolysaccharide (LPS) was used to induce cell pyroptosis in an animal model of pulmonary fibrosis. After treatment with diazepam, changes in cell proliferation and apoptosis were observed, and the occurrence of inflammation and pulmonary fibrosis in the mice was detected.

**Results:**

The results showed that LPS can successfully induce cell pyroptosis and inflammatory responses and cause lung fibrosis in mice. Diazepam inhibits the expression of pyroptosis-related factors and inflammatory factors; moreover, it attenuates the occurrence of pulmonary fibrosis in mice. Mechanistically, diazepam can upregulate the expression of let-7a-5p, inhibit the expression of MYD88, and reduce inflammation and inhibit pulmonary fibrosis by regulating the let-7a-5p/MYD88 axis.

**Conclusion:**

Our findings indicated that diazepam can inhibit LPS-induced pyroptosis and inflammatory responses and alleviate pulmonary fibrosis in mice by regulating the let-7a-5p/MYD88 axis.

## 1 Introduction

Pulmonary fibrosis (PF) is a progressively fatal disease characterized by massive proliferation of lung fibroblasts, accumulation of extracellular matrix, massive deposition of collagen matrix and the production of proinflammatory cytokines [1,2]. In recent years, its morbidity has gradually increased; moreover, its prognosis is poor, and its mortality rate is high [3,4]. There is currently no convenient or effective prevention or treatment method for pulmonary fibrosis. Early drugs that are used to treat pulmonary fibrosis, such as glucocorticoids, cyclosporine A, and cyclophosphamide, have unsatisfactory curative effects and severe side effects; however, the efficacy and safety of the new drugs pirfenidone and nintedanib for long-term use have not been determined [5,6]. Therefore, it is highly important to explore potential pulmonary fibrosis treatment drugs. Traditionally, pulmonary fibrosis is a chronic inflammatory-related response, and the stress response produced by alveolar epithelial cells after damage involves an inflammatory response. Inflammatory factors can directly act on alveoli to aggravate damage and cause a vicious cycle to occur by activating related inflammatory cells and lymphocytes [7]. Therefore, based on its anti-inflammatory effects, we are seeking potential treatment drugs for pulmonary fibrosis and exploring the underlying mechanism.

Diazepam is a benzodiazepine that has an effect on immune cells [8]. Diazepam can inhibit T-cell function by reducing cell proliferation and the production of proinflammatory cytokines (IFN-γ and IL-17) [9,10]. In addition, diazepam can also reduce the release of proinflammatory cytokines (TNF-α, IL-6, and IL-12) by affecting innate immune cells [11]. Diazepam has a protective effect on the development of experimental autoimmune encephalomyelitis by reducing the incidence of the disease by reducing the number of inflammatory cells in the central nervous system; furthermore, diazepam can reduce the levels of the biomarker transposon protein translocator protein (TSPO), which is a biomarker of inflammatory diseases in brain tissue and inflammatory CD11b^+^ cells in the central nervous system [12]. Diazepam regulates the immune system and inflammation and may be a potential therapeutic drug for the treatment of pulmonary fibrosis in the clinical setting. Therefore, this study investigated the effect of diazepam on pulmonary fibrosis.

Pyroptosis, which is an inflammatory form of cell death triggered by certain inflammasomes, is strongly associated not only with the proliferation and migration of a variety of cancers [13] but also with different pathophysiological outcomes in chronic inflammatory diseases [14]. Pyroptosis also plays an important role in the development of lung diseases by inhibiting the activation of Nod-like receptor pyrin domain 3 (NLRP3) inflammasomes, which can alleviate lung injury induced by phosgene [15]. Studies have also shown that NLRP3 inflammasome-mediated cell pyroptosis is closely related to idiopathic pulmonary fibrosis (IPF). Studies have also shown that NLRP3 inflammasome-mediated pyroptosis is closely related to idiopathic pulmonary fibrosis, and NLRP3 is activated to recruit more inflammatory cells by releasing proinflammatory factors to enhance the local inflammatory response, thus inducing idiopathic pulmonary fibrosis [16]. Lipopolysaccharide (LPS) can trigger pyroptosis and promote the secretion of proinflammatory cytokines by initiating and activating NLRP3 [17]. LPS also induces the death of macrophages and cancer cells through the caspase-11-dependent pyroptosis pathway [18] and can also induce pyroptosis to promote acute lung injury and fibrosis [19]. For this reason, this study investigated whether diazepam has an inhibitory effect on LPS-induced pyroptosis and inflammation to determine its ability to alleviate pulmonary fibrosis.

MicroRNAs (miRNAs) are small noncoding RNA molecules approximately 22 nucleotides in length that can induce mRNA silencing and posttranscriptional inhibition to affect gene expression [20]. MiRNAs have been found to be related to the pyroptosis of specific cancers. For example, miRNA-214 was found to induce pyroptosis by directly targeting caspase-1 through the cleavage of Gasdermin D (GSDMD) [21,22]. The let-7 family is a group of miRNAs that have been well studied in many diseases and are closely related to lung cancer. For example, the downregulation of let-7a-2 is associated with poor lung cancer survival; moreover, let-7 has been found to inhibit the growth of multiple human lung cancer cell lines in vitro [23], and let-7a-5p synergistically inhibits lung cancer cell proliferation by inducing G1/S phase arrest [24]. Additionally, the expression of liver let-7a-5p is downregulated in cell carcinoma [25], and its role in liver fibrosis has also been determined; specifically, the overexpression of let-7a-5p can inhibit the LPS-induced muscle activation of cultured human hepatic stellate cells [26]. However, the effect of let-7a-5p on pulmonary fibrosis has not been reported; thus, we investigated whether let-7a-5p affects the progression of pulmonary fibrosis by regulating pyroptosis. Myeloid differentiation primary response protein 88 (MYD88) is a key adaptor protein involved in the interleukin and Toll-like receptor signal transduction pathways that controls the innate immune response and inflammation. It can form an activated downstream kinase cascade with a MyD88 dimer in the Toll/interleukin-1 receptor (TIR) domain, thus ultimately leading to the production of inflammatory mediators [27,28], and it can be used as a therapeutic target for inflammatory lung diseases [29]. Bioinformatics software has predicted that let-7a-5p and MYD88 have targeted binding sites. For this reason, let-7a-5p is proposed to regulate MYD88 expression to affect cell pyroptosis.

In this study, we investigated the inhibitory effect of diazepam on the progression of pulmonary fibrosis. We further confirmed whether diazepam can alleviate the progression of pulmonary fibrosis by regulating the let-7a-5p/MYD88 axis through cell pyroptosis and the inflammatory response. Our research provides a potential clinical therapeutic drug and a new potential therapeutic target for pulmonary fibrosis treatment.

## 2 Materials and methods

### 2.1 Animal experiment

Forty-five healthy 8–10-week-old male C57BL/6 mice weighing approximately 20–24 g were raised in Specific pathogen free (SPF) independent isolation cages in the Experimental Animal Center. The feed was strictly sterilized, and the experiment was performed after 4–5 days of adaptive feeding. The mice were randomly divided into five groups. In the LPS group (n = 15), 1.5 mg/kg LPS was injected into the abdominal cavity of the mice. For the control group (n = 10), the mice were intraperitoneally injected with the same amount of normal saline. In the LPS+Diazepam group (n = 10), after LPS injection for 2 h, the mice were intraperitoneally injected with diazepam (0.7 mg/10 μL/g b.w.). In the LPS+Diazepam+ let-7a-5p inhibitor group (n = 5), the mice were intraperitoneally injected with diazepam or the let-7a-5p inhibitor through the tail vein. In the LPS+Diazepam+ OE-MYD88 group (n = 5), the mice were intraperitoneally injected with diazepam or OE-MYD88 through the tail vein. During the experiment, the mice exhibited pain or painful behaviors, and the mice were given buprenorphine (0.1 mg/kg) for analgesia. On the 7th day after LPS treatment, the mice were sacrificed via cervical dislocation. Mouse serum and lung tissue were collected, and the expression levels of related genes in the serum and lung tissue were detected. All of the animals were treated in accordance with the Guide for the Care and Use of Laboratory Animals, and the treatment was approved by the Ethics Committee of Kunming Medical University (No. Kmmu20211589).

### 2.2 Cell culture

Human bronchial epithelioid cells (HTX2577, 16HBE) and human normal lung bronchial epithelial cells (HTX2075, BEAS-2B) were purchased from Otwo Biotech. 16HBE and BEAS-2B cells were cultured in Dulbecco’s modified Eagle’s medium (DMEM) supplemented with 10% fetal bovine serum. The plates were placed in an incubator at 37°C and 5% CO_2_. Enzyme pancreatic digestion was used for subsequent experiments when the cells grew to the logarithmic growth phase. The cells were exposed to LPS (100, 200, or 500 ng/mL) for 24 hours. After 2 hours of LPS treatment, the cells were treated with diazepam (1 μM) for 12 hours.

### 2.3 RT‒qPCR

According to the instructions, the RNA extraction reagent TRIzol (Takara, Tokyo, Japan) was used to extract total RNA from 16HBE and BEAS-2B cells. Afterwards, the RNA was reverse transcribed into complementary DNA (cDNA) according to the PrimeScript™ RT kit (Takara, Tokyo, Japan). RT‒qPCR was performed by using a SYBR Premix Ex Taq Kit (Takara, Tokyo, Japan). A CFX96 real-time fluorescent quantitative PCR assay system (Bio-Rad, Hercules, CA, USA) was used for fluorescence quantitative PCR. The relative expression was calculated by using the 2^-ΔΔCt^ method with U6 as the internal reference. The utilized primers are listed in Table 1.

**Table 1:** Primer sequence.

Gene	Forward sequence	Reverse sequence
let-7a-5p	5’-GCGCGTGAGGTAGTAGGTTGT-3’	5’- AGTGCAGGGTCCGAGGTATT -3’
U6	5’- CTCGCTTCGGCAGCACA -3’	5’- AACGCTTCACGAATTTGCGT -3’

### 2.4 Western blot

A protein extraction kit (Cat# KGP2100, KeyGEN BioTECH, Jiangsu, China) was used to extract total protein from 16HBE and BEAS-2B cells and mouse lung tissue. A BCA protein quantification kit was used to determine the protein concentration. Subsequently, the proteins were separated by using sodium dodecyl sulfate‒polyacrylamide gel electrophoresis (SDS‒PAGE), transferred to polyvinylidene fluoride (PVDF) membranes, and incubated with primary antibodies against Bcl-2 (ab182858, 1:2000, Abcam, UK), Caspase-3 (ab13847, 1:500, Abcam, UK), Bax (ab53154, 1:1000, Abcam, UK), Caspase1 (ab138483, 1:1000, Abcam, UK), Gasdermin D (ab155233, 1:1000, Abcam, UK), IL-1β (ab254360, 1:1000, Abcam, UK), IL-4 (ab34277, 1:1000, Abcam, UK), IL-6 (ab233706, 1:1000, Abcam, UK), and TNF-α (ab183218, 1:1000, Abcam, UK) overnight at 4°C. Afterwards, the PVDF membrane was incubated with a secondary antibody. The color was developed by using an enhanced chemiluminescence (ECL) kit (Millipore, USA). Finally, ImageJ software was used to conduct a semiquantitative analysis of the bands.

### 2.5 MTT detection of cell proliferation

The cells were inoculated in a 96-well plate (2000 cells/well, 200 μL), the cell culture time was recorded as 0 h when observing the adherence of the cells, 0.02 mL of MTT reagent was added, and the plates were incubated in an incubator (37°C, protected from light) for 4 hours. Afterwards, the MTT reagent was added, 0.15 mL of DMSO was added, the mixture was incubated for 15 minutes, the solution was mixed well, and the optical density (OD) was measured with a 570 nm microplate reader. To ensure the accuracy of the experimental results, the MTT experiment was repeated 3 times.

### 2.6 Flow cytometry

The cells were collected, 1×10^6^ cells were resuspended in 200 μL of binding buffer, 4 μL of 0.5 mg/mL PI and 2 μL of Annexin V-FITC solution were added, and the mixture was incubated for 15 min at room temperature in the dark. Apoptosis was detected by flow cytometry.

### 2.7 Masson staining

The collected lung tissue was first fixed with 4% paraformaldehyde. The tissues were then dehydrated with different concentrations of ethanol and xylene. The dehydrated tissue was embedded after wax immersion and subsequently sectioned. The tissue sections were immersed in hematoxylin staining solution for 5 minutes, washed with distilled water, differentiated with 5% acetic acid for 1 minute, and washed with distilled water for 5 minutes. The sections were immersed in Masson staining solution for 5–10 minutes, rinsed with water, and soaked in 1% phosphotungstic acid solution for 5 minutes. The samples were then placed in a dyeing tank filled with aniline blue solution for 5 min, after which glacial acetic acid water (1% concentration) was added dropwise for 2 min. The samples were soaked in 70%, 80%, 90%, and 100% ethanol for 10 seconds and in xylene for 1 minute, after which they were allowed to dry naturally in a fume hood. A pipette was used to add 1–2 drops of rhamsan gum, and the mixture was covered with a cover glass and pressed to ensure that the tissue was completely covered and to avoid for formation of air bubbles in the middle of the glass.

### 2.8 Statistical analysis

All of the statistical analyses were performed by using GraphPad Prism 8.0 (GraphPad Software Inc., San Diego, CA, USA). The statistical results are expressed as the mean ± standard deviation (SD). An unpaired t test was used for comparisons between two groups, and one-way analysis of variance (ANOVA) was used for comparisons between different groups. Both P<0.05 and P<0.01 indicate statistically significant differences.

## 3 Results

### 3.1 Effect of LPS on the proliferation and apoptosis of 16HBE and BEAS-2B cells

**Fig 1:**
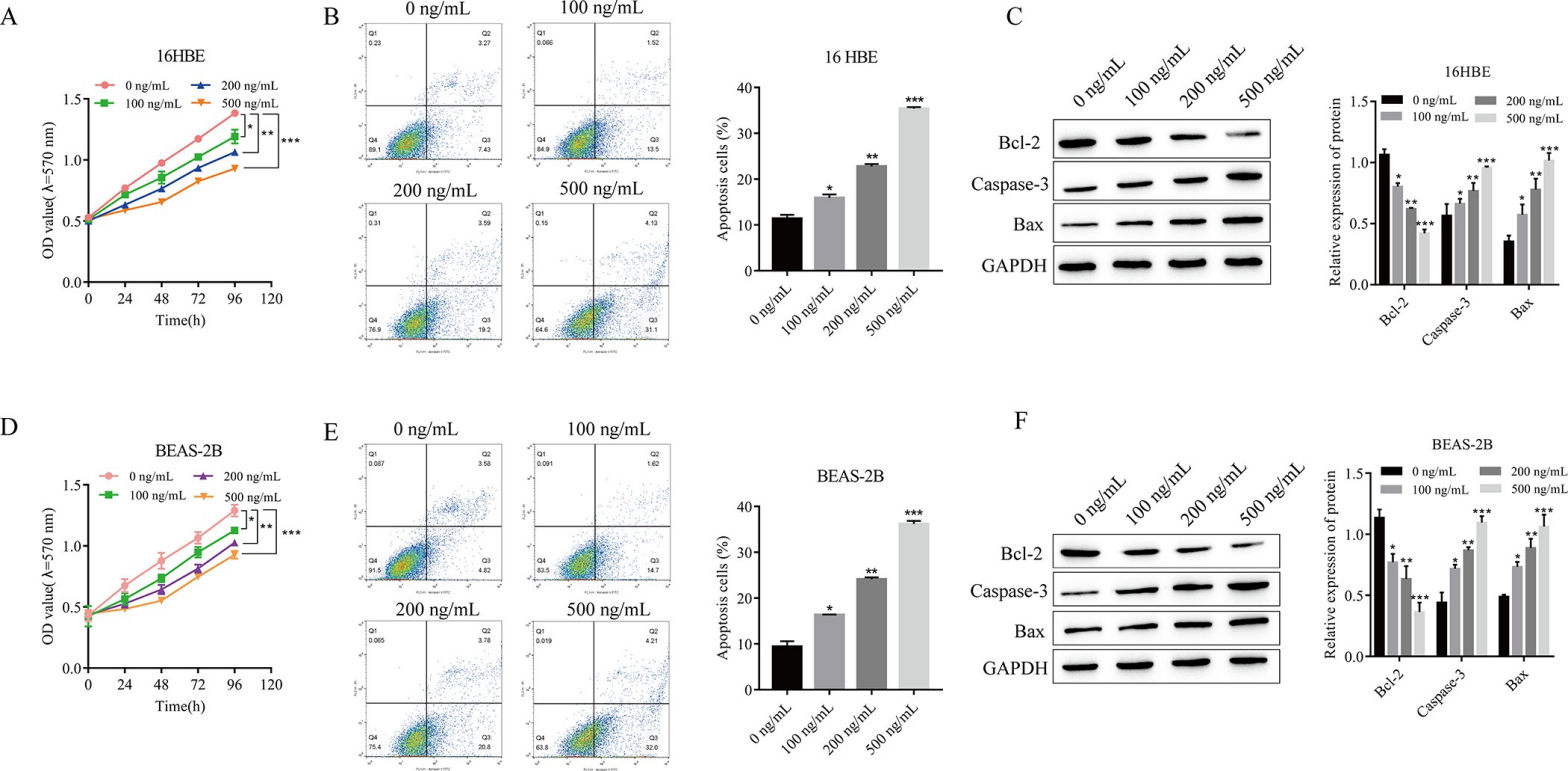
Effect of LPS on the proliferation and apoptosis of 16HBE and BEAS-2B cells. (A) The proliferation of 16HBE cells was detected by using MTT. (B) Apoptosis of 16HBE cells was detected by using flow cytometry. (C) The expression of apoptosis-related proteins in 16HBE cells was detected via Western blotting. (D) The proliferation of BEAS-2B cells was detected by using MTT. (E) The apoptosis of BEAS-2B cells was detected by using flow cytometry. (F) The expression of apoptosis-related proteins in BEAS-2B cells was detected via Western blotting. *P<0.05, **P<0.01, ***P<0.001 vs. 0 ng/mL. (A-F: n = 3).

LPS is an ideal drug for inducing pulmonary fibrosis. Therefore, we treated 16HBE and BEAS-2B cells with different concentrations of LPS (0, 100, 200, and 500 ng/mL). In 16HBE cells, after LPS treatment, cell proliferation was significantly decreased (Fig 1A), and apoptosis was significantly increased (Fig 1B); both of these effects occurred in an LPS concentration-dependent manner. Western blot results showed that after LPS treatment, the expression of the antiapoptotic protein Bcl-2 was significantly decreased, the expression of the apoptotic proteins Caspase-3 and Bax was significantly increased, and the changes in protein expression gradually increased with increasing LPS concentration (Fig 1C). Similarly, in BEAS-2B cells, LPS significantly reduced proliferation and promoted apoptosis in a dose-dependent manner (Fig 1D–1F). Due to the fact that the effect of LPS treatment (500 ng/mL) was the most obvious, 16 HBE and BEAS-2B cells were treated with a concentration of LPS (500 ng/mL) in subsequent experiments.

### 3.2 LPS induced pyroptosis and inflammation in 16HBE and BEAS-2B cells and promoted the progression of pulmonary fibrosis

**Fig 2:**
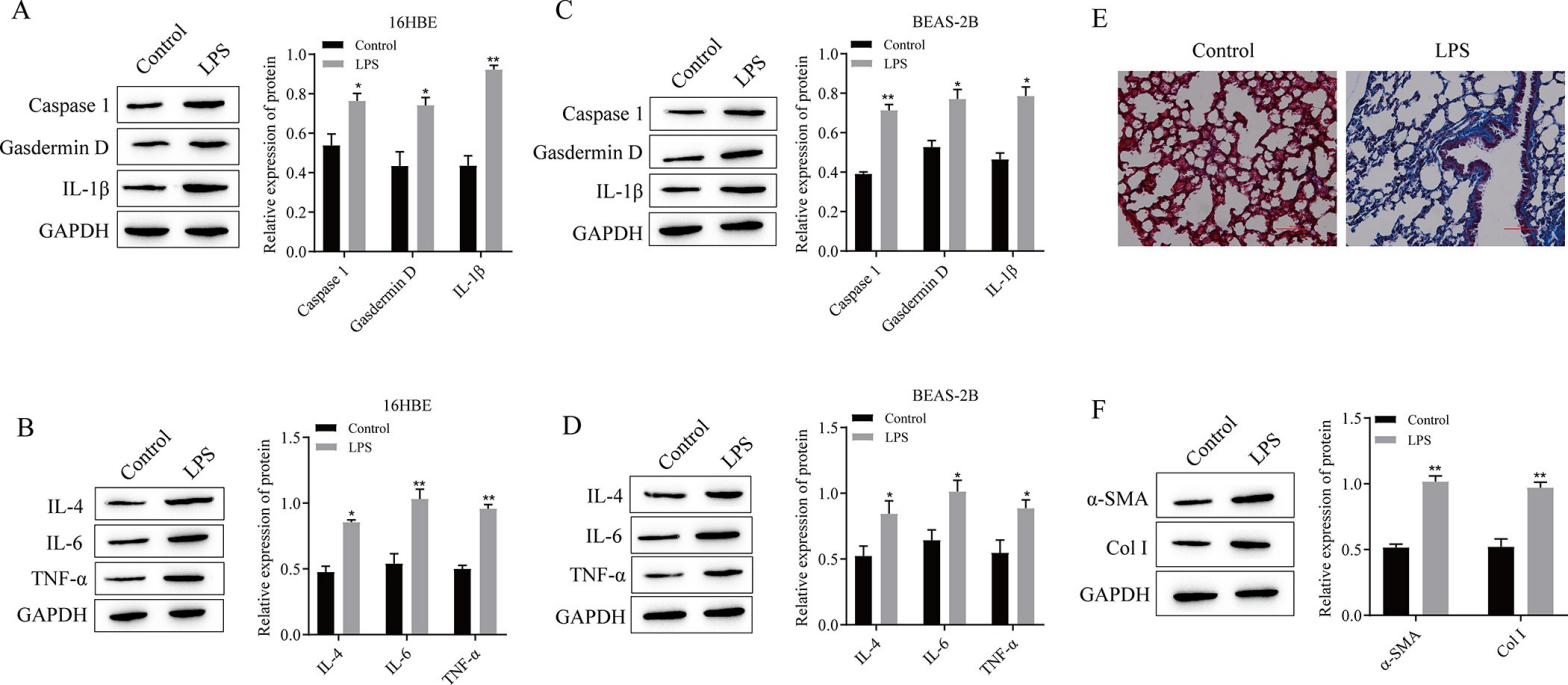
LPS induced pyroptosis and inflammation in 16HBE and BEAS-2B cells and promoted the progression of pulmonary fibrosis. (A) Western blotting was used to detect the expression of pyroptosis-related proteins in 16HBE cells. (B) Western blotting was used to detect the expression of inflammatory factors in 16HBE cells. (C) Western blotting was used to detect the expression of pyroptosis-related proteins in BEAS-2B cells. (D) Western blotting was used to detect the expression of inflammatory cytokines in BEAS-2B cells. (E) Masson staining was used to detect the degree of pulmonary fibrosis. (F) The expression of the pulmonary fibrosis-related proteins α-SMA and Col I was detected via Western blotting. *P<0.05, **P<0.01 vs. Control. (A-D: n = 3; E-F: n = 5).

The dose-dependent effects of LPS on the proliferation and apoptosis of 16HBE and BEAS-2B cells were confirmed, and the effects of LPS on pyroptosis, inflammation and fibrosis progression were confirmed. We treated 16HBE and BEAS-2B cells with 500 ng/mL LPS and induced pulmonary fibrosis in the mice by injecting 1.5 mg/kg LPS. First, the results of cell experiments showed that the expression of the pyroptosis-related proteins Caspase1, Gasdermin D, and IL-1β was significantly greater in the LPS-induced group than in the control group, and the expression of the inflammatory cytokines IL-4, IL-6, and TNF-α was significantly upregulated (Fig 2A–2D). The results of Masson staining of mouse lung tissue showed that compared with those in the control group, a large number of collagen fibers that were stained blue appeared in the LPS-induced group, thus indicating severe fibrosis (Fig 2E). Moreover, Western blot analysis of the expression of fibrosis-related proteins demonstrated that the expression of α-SMA and Col I in the LPS group was significantly greater than that in the control group (Fig 2F).

### 3.3 Diazepam alleviates LPS-induced pyroptosis, inflammation and the progression of pulmonary fibrosis

**Fig 3:**
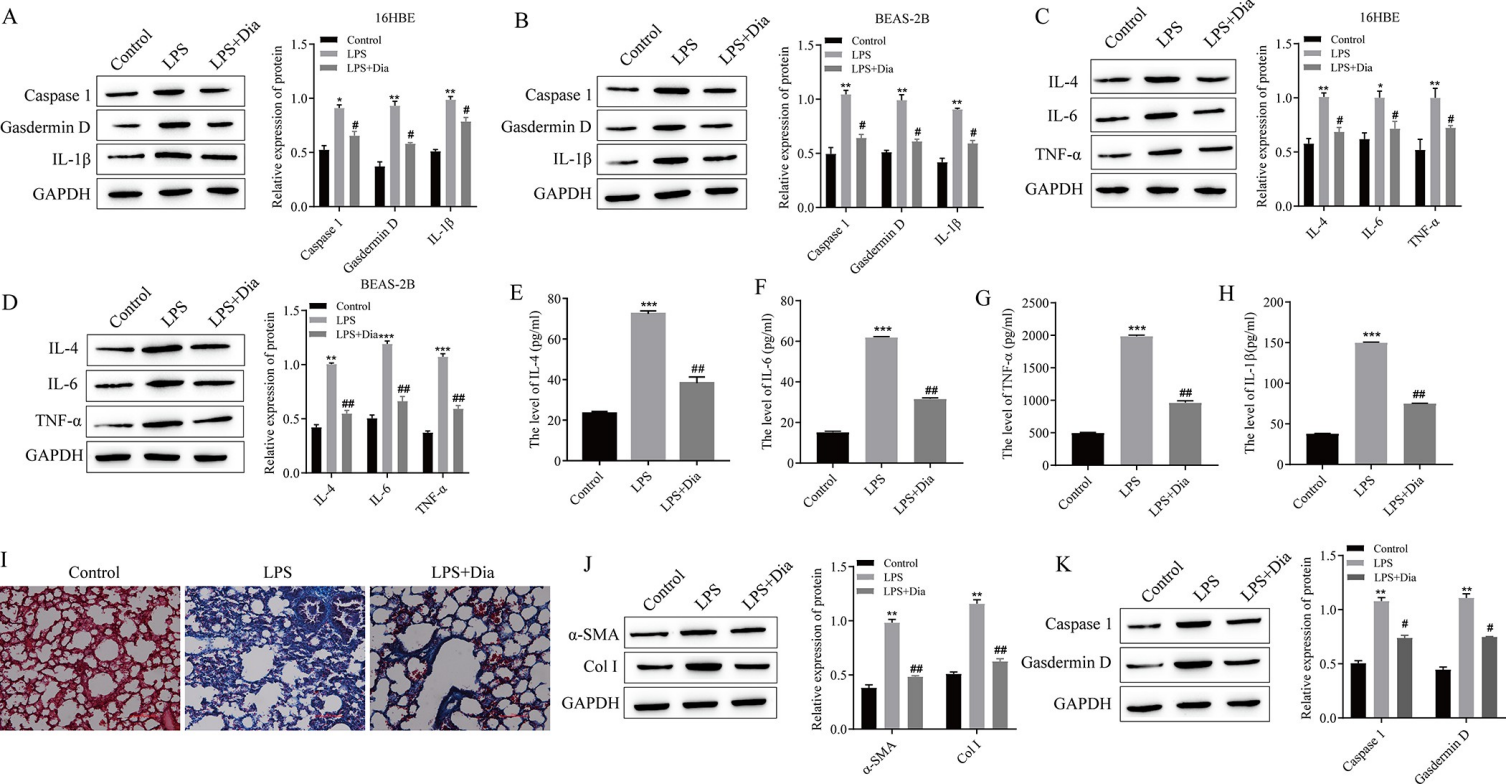
Diazepam alleviates LPS-induced pyroptosis, inflammation and the progression of pulmonary fibrosis. (A) Western blotting was used to detect the expression of pyroptosis-related proteins in 16HBE cells. (B) Western blotting was used to detect the expression of pyroptosis-related proteins in BEAS-2B cells. (C) Western blotting was used to detect the expression of inflammatory factors in 16HBE cells. (D) Western blotting was used to detect the expression of inflammatory factors in BEAS-2B cells. (E) The expression of IL-4 was detected by using ELISA. (F) The expression of IL-6 was detected via ELISA. (G) The expression of TNF-α was detected by using ELISA. (H) The expression of IL-1β was detected by using ELISA. (I) Masson staining was used to detect the degree of pulmonary fibrosis. (J) Western blotting was used to detect the expression of the pulmonary fibrosis-related proteins α-SMA and Col I in mouse lung tissue. (K) Western blotting was used to detect the expression of pyroptosis-related proteins in mouse lung tissue. *P<0.05, **P<0.01, ***P<0.001 vs. Control; #P<0.05, ##P<0.01 vs. LPS. (A-H: n = 3; I-K: n = 5).

Diazepam was administered after LPS treatment of 16HBE and BEAS-2B cells or to induce pulmonary fibrosis in mice. The results showed that in 16HBE and BEAS-2B cells, compared with those in the LPS group, the expression levels of the pyroptosis-related proteins Caspase1, Gasdermin D and IL-1β were significantly decreased in the LPS+Diazepam(LPS+Dia) group, and the expression levels of the inflammatory cytokines IL-4, IL-6 and TNF-α were also significantly downregulated (Fig 3A–3D). The expression of inflammatory factors in the serum of mice was detected via ELISA, and the results showed that the levels of IL-4, IL-6, TNF-α and IL-1β were significantly lower in the LPS+Diazepam group than in the LPS group (Fig 3E–3H). Masson staining demonstrated that fibrosis was significantly reduced in the LPS+Diazepam group compared with the LPS group (Fig 3I). Moreover, Western blotting was used to detect the expression of pulmonary fibrosis-related proteins and pyroptosis-related proteins in lung tissues. The protein expression of α-SMA, Col I, Caspase1 and Gasdermin D in the diazepam treatment group was significantly downregulated (Fig 3J–3K).

### 3.4 Diazepam regulates LPS-induced pyroptosis and inflammation via the let-7a-5p/MYD88 axis

**Fig 4:**
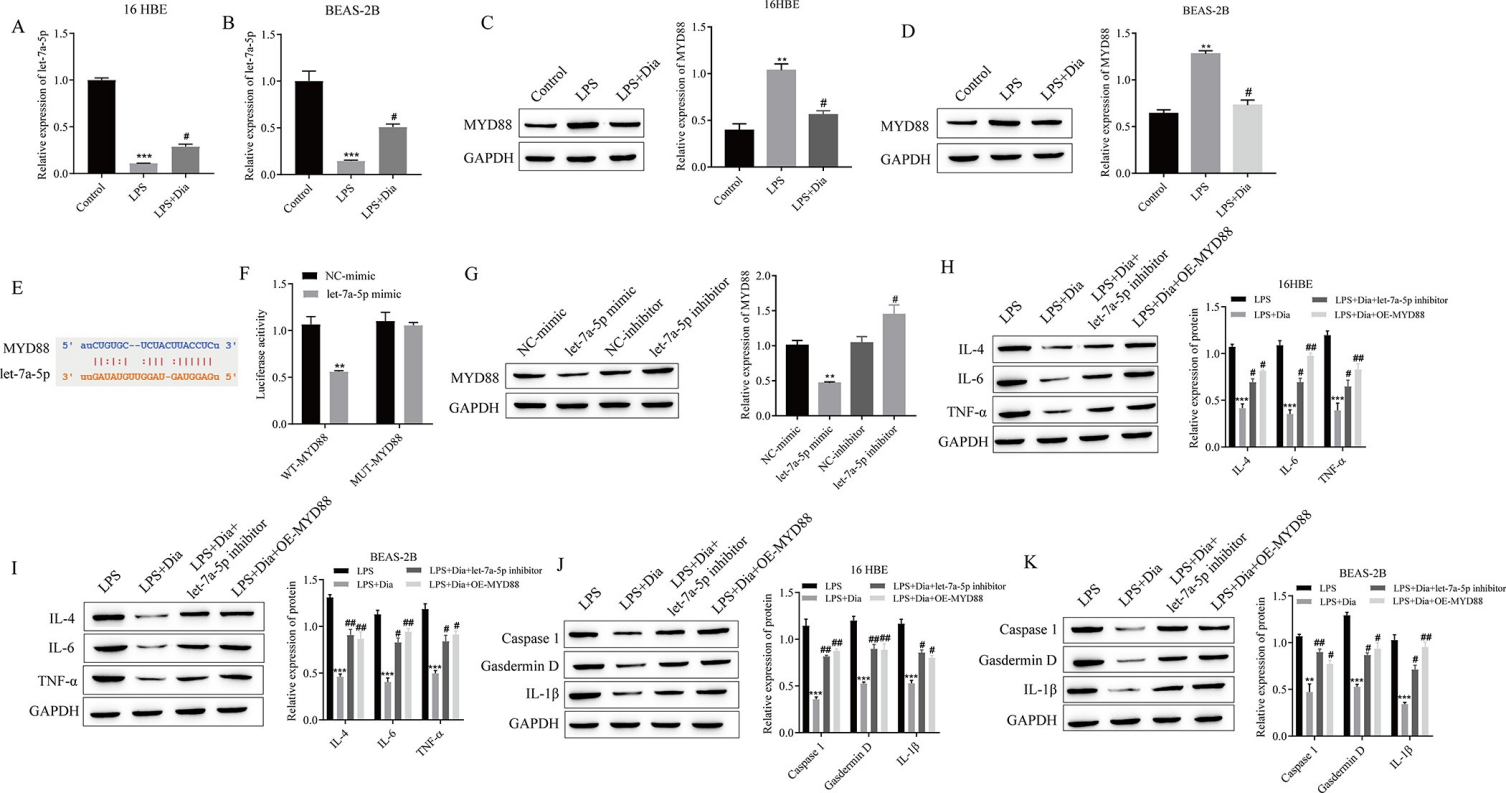
Diazepam regulates LPS-induced pyroptosis and inflammation via the let-7a-5p/MYD88 axis. The expression of let-7a-5p in 16HBE cells was detected by using RT‒qPCR. (B) The expression of let-7a-5p in BEAS-2B cells was detected by using RT‒qPCR. (C) Western blotting was used to detect the protein expression of MYD88 in 16HBE cells. (D) The protein expression of MYD88 in BEAS-2B cells was detected via Western blotting. (E) Prediction of let-7a-5p and MYD88 binding sites. (F) Double luciferase gene reporting experiment. (G) The expression of MYD88 was detected by using Western blotting. (H) Western blotting was used to detect the expression of inflammatory factors in 16HBE cells. (I) Western blotting was used to detect the expression of inflammatory factors in BEAS-2B cells. (J) Western blot analysis of pyroptosis-related protein expression in 16HBE cells. (K) Western blotting was used to detect the expression of pyroptosis-related proteins in BEAS-2B cells. **P<0.01, ***P<0.001 vs. Control, NC-mimic or LPS; #P<0.05, ##P<0.01 vs. LPS, NC-inhibitor or LPS+Dia. (A-K: n = 3).

We further explored the mechanism by which diazepam inhibits LPS-induced pyroptosis and the inflammatory response. We found that LPS could significantly reduce the expression of let-7a-5p and significantly increase the expression of MYD88, whereas the addition of diazepam could reverse the abovementioned phenomena, to some extent (Fig 4A–4D). A targeted binding site between let-7a-5p and MYD88 was predicted by using the StarBase website (Fig 4E). The dual-luciferase gene reporter assay further confirmed the targeted binding relationship between let-7a-5p and MYD88 (Fig 4F). Western blotting confirmed that let-7a-5p negatively regulates the expression of MYD88 (Fig 4G). 16HBE and BEAS-2B cells were transfected with a let-7a-5p inhibitor or an MYD88 overexpression (OE-MYD88) plasmid to observe the effects of diazepam on these cells. Western blot analysis of inflammation- and apoptosis-related protein expression showed that let-7a-5p inhibitior or OE-MYD88DE transfection significantly reversed the inhibitory effect of diazepam on the protein expression of IL-4, IL-6, TNF-α, Caspase1, Gasdermin D and IL-1β (Fig 4H–4K).

### 3.5 Diazepam attenuated the effects of LPS on cell proliferation and apoptosis and alleviated pulmonary fibrosis through the let-7a-5p/MYD88 axis

**Fig 5:**
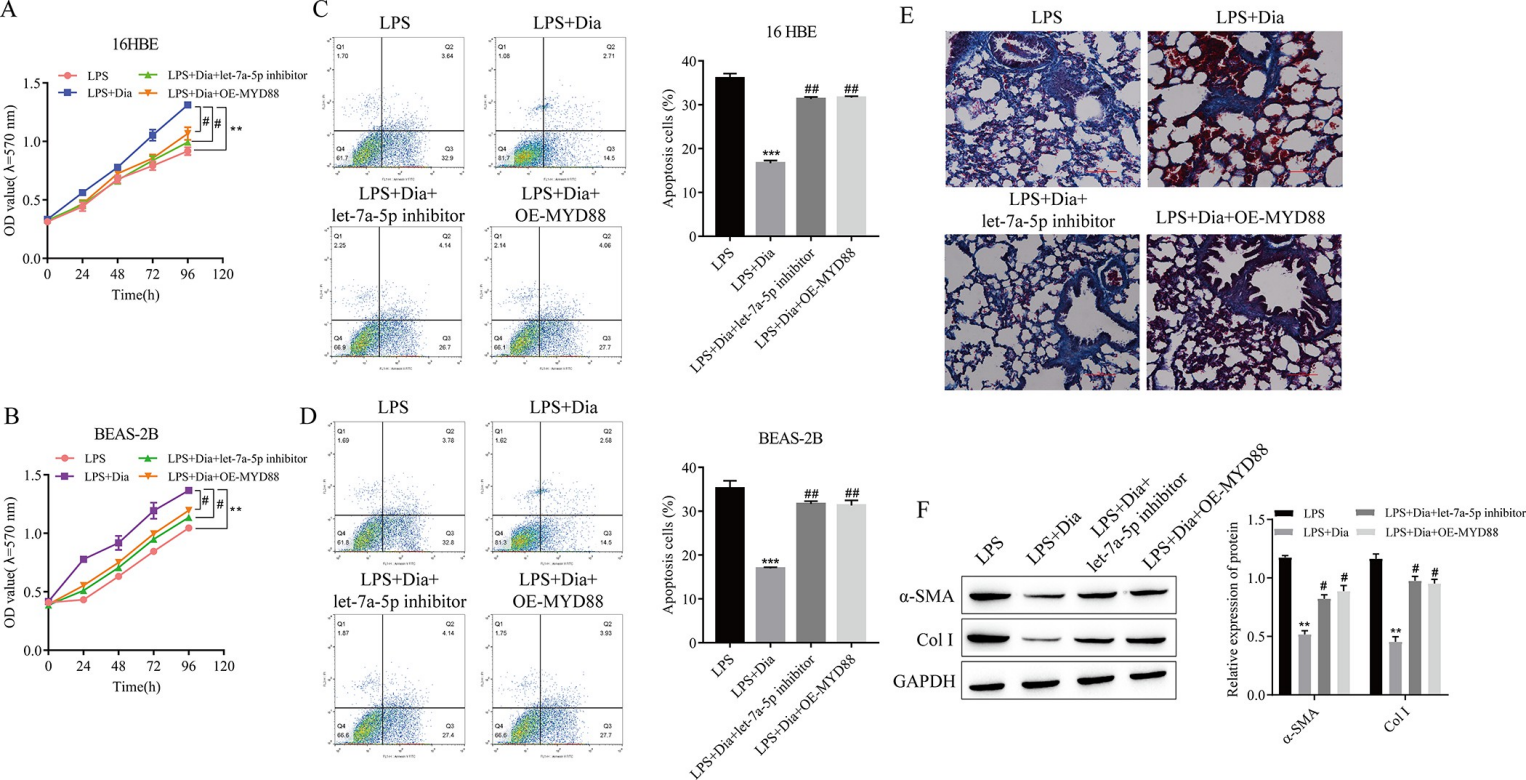
Diazepam attenuated the effects of LPS on cell proliferation and apoptosis and alleviated the occurrence of pulmonary fibrosis through the let-7a-5p/MYD88 axis. (A) The proliferation of 16HBE cells was detected by using MTT. (B) The proliferation of BEAS-2B cells was detected by using MTT. (C) The apoptosis of 16HBE cells was detected by using flow cytometry. (D) The apoptosis of BEAS-2B cells was detected by using flow cytometry. (E) Masson staining was used to detect the degree of pulmonary fibrosis. (F) The expression of the pulmonary fibrosis-related proteins α-SMA and Col I was detected via Western blotting. **P<0.01, ***P<0.001 vs. LPS; #P<0.05, ##P<0.01 vs. LPS+Dia. (A-D: n = 3; E-F: n = 5).

The results of cell proliferation and apoptosis showed that compared with those in the LPS group, the proliferation of 16HBE and BEAS-2B cells was significantly increased, and apoptosis was significantly decreased, in the LPS+Diazepam group. However, compared with those in the LPS+Diazepam group, the proliferation of 16HBE and BEAS-2B cells in the LPS+Diazepam+let-7a-5p inhibitor or LPS+Diazepam+OE-MYD88 group was significantly reduced, and apoptosis was significantly increased (Fig 5A–5D). The results of Masson staining of mouse lung tissue showed that the inhibitory effect of diazepam on pulmonary fibrosis was significantly reversed by treatment with the let-7a-5p inhibitor or OE-MYD88 (Fig 5E). The inhibitory effect of diazepam on the expression of the fibrosis-related proteins α-SMA and Col I was also significantly reversed by the let-7a-5p inhibitor or OE-MYD88 (Fig 5F).

## 4 Discussion

Pulmonary fibrosis is caused by progressive dyspnea, wheezing, shortness of breath, dry cough and other clinical manifestations; moreover, it is characterized by restrictive ventilatory dysfunction, hypoxemia, and chronic progressive and diffuse pulmonary interstitial fibrosis. The etiology of pulmonary fibrosis in modern medicine is not clear. In addition, there is still no effective treatment for pulmonary fibrosis due to its complex pathogenesis [30]. Therefore, the identification of the mechanism of pulmonary fibrosis and the discovery of new potential therapeutic drugs for promoting effective treatment of pulmonary fibrosis are highly important. LPS-induced cells and mouse pulmonary fibrosis cell models are relatively mature; therefore, this study used LPS-induced methods to simulate the PF process. Consistent with previous studies [31], in the present study, LPS induced pyroptosis and inflammation in 16HBE and BEAS-2B cells and successfully induced pulmonary fibrosis in mice. Moreover, the effects of LPS on the proliferation and apoptosis of 16HBE and BEAS-2B cells were confirmed in this study, which is consistent with the findings of previous studies showing that LPS-mediated inhibition of cell proliferation and promotion of apoptosis are critical for the progression of pulmonary fibrosis [32].

Studies have shown that acute lung injury is related to pyroptosis [15], and diffuse alveolar damage caused by the progression of acute lung injury may lead to pulmonary fibrosis [33]. Recent studies have also shown that pyroptosis is closely related to idiopathic pulmonary fibrosis (IPF), and the increased expression of IL-1β and IL-18 in the serum and lung tissues of mice induced by bleomycin was observed in the IPF model [34]; moreover, pyroptosis is the direct cause of the increased expression of IL-1β and IL-18 in lung tissues [35]. It has been reported that the application of pyroptosis inhibitors can reduce the inflammatory response of lung tissues and inhibit pulmonary fibrosis [36]. Therefore, pyroptosis plays an important role in pulmonary fibrosis. This study confirmed that LPS-induced pulmonary fibrosis is associated with pyroptosis and that LPS can promote the expression of the pyroptosis-related factors Caspase1, Gasdermin D and IL-1β.

In the early stage of pulmonary fibrosis, a strong inflammatory response occurs in lung tissue, which is manifested as a large amount of inflammatory cell infiltration; additionally, the inflammatory response of the lung tissue is highly important for the development of pulmonary fibrosis [37]. Under conditions of lung injury, inflammation and the immune response promote the formation of IPF, and cytokines that are involved in this process, such as TGF-β, TNF-α, IL-1β and IL-6, cause inflammatory cells to infiltrate, differentiate and undergo maturation, and promote further secretion of inflammatory cytokines and inflammatory transmitters by these cells, thus resulting in alveolitis and further lung injury [38]. Hypnotic anesthetics have been proven to effectively regulate inflammatory responses in a variety of diseases [39]; therefore, they are an ideal drug for the treatment of pulmonary fibrosis. Diazepam inhibits inflammation during influenza infection [40]. In this study, we verified that diazepam can effectively inhibit inflammation in pulmonary fibrosis; specifically, diazepam can effectively inhibit the expression of the inflammatory cytokines IL-4, IL-6 and TNF-α and effectively alleviate the occurrence of pulmonary fibrosis. Diazepam can also eliminate LPS-induced pyroptosis. This finding is consistent with the results of our other research. In previous studies, we found that diazepam alleviated LPS-induced pyrodeath and the development of pulmonary fibrosis in mice by activating α4-GABA_A_Rs [41]. In this study, we demonstrated a new mechanism by which diazepam alleviates pulmonary fibrosis.

We found that let-7a-5p expression was significantly downregulated after LPS treatment in 16HBE and BEAS-2B cells, which was consistent with the significantly downregulated let-7a-5p expression observed in the lung tissue of a mouse model of acute respiratory distress syndrome [42]. Previous studies have shown that TGF-β1 is a key cytokine in fibrosis [43] and that inhibitors targeting TGF-β1 signaling and its receptors can reduce bleomycin-induced pulmonary fibrosis in mice [44]. Therefore, abnormal let-7a-5p expression is an important factor in the occurrence of pulmonary fibrosis. MYD88 is a key downstream molecule of the Toll-like receptor inflammatory signaling pathway [45]. The MYD88-dependent signaling pathway activates NF-κB, thus resulting in inflammatory cytokine release [46]. The IL-1 family is a key cytokine that is downstream of MYD88, and its members have potential proinflammatory effects and can participate in inflammatory reactions [47]. It has been reported that the expression of MYD88 and IL-1β is increased in bleomycin-induced pulmonary fibrosis in mice [36]. This study also showed that the expression of MYD88 and IL-1β significantly increased in LPS-induced pulmonary fibrosis, whereas the overexpression of let-7a-5p significantly downregulated the expression of MYD88. Moreover, a targeted binding site between let-7a-5p and MYD88 was identified. In addition, diazepam significantly upregulated let-7a-5p expression and significantly inhibited MYD88 expression. When let-7a-5p expression was inhibited or MYD88 was overexpressed, the inhibitory effect of diazepam on LPS-induced cell pyroptosis and inflammation was considerably weakened, and the same was true for the alleviation of fibrosis. Therefore, this study demonstrated that diazepam can upregulate the expression of let-7a-5p and inhibit the expression of MYD88 to reduce pyroptosis and inflammation, thus ultimately alleviating the progression of pulmonary fibrosis.

## 5 Conclusions

In conclusion, we demonstrated that diazepam inhibits LPS-induced pyroptosis and inflammation and reduces the incidence of pulmonary fibrosis in mice by regulating the let-7a-5p/MYD88 axis. This study can increase the selection of diazepam for drug screening in the treatment of pulmonary fibrosis and provide new potential therapeutic targets for let-7a-5p and MYD88 for molecular target treatment of pulmonary fibrosis.

## Supporting information

S1 Raw images (PDF)
