# Supplementary material for: Diazepam inhibits LPS-induced pyroptosis and inflammation and alleviates pulmonary fibrosis in mice by regulating the let-7a-5p/MYD88 axis
Source: PLoS One. 2024 Jun 14;19(6):e0305409. doi: 10.1371/journal.pone.0305409 (PMC11178199; doi:10.1371/journal.pone.0305409)

Figure 1C

Bcl-2 (26 kDa)

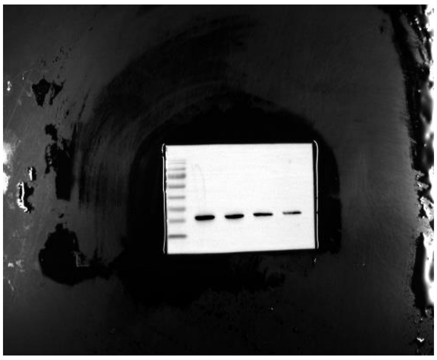

Caspase-3 (17 kDa)

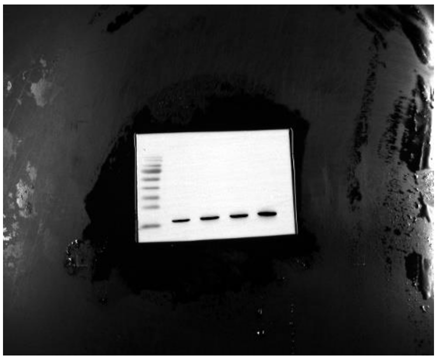

Bax (21 kDa)

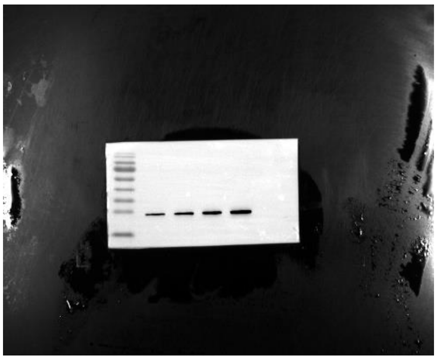

GAPDH (37 kDa)

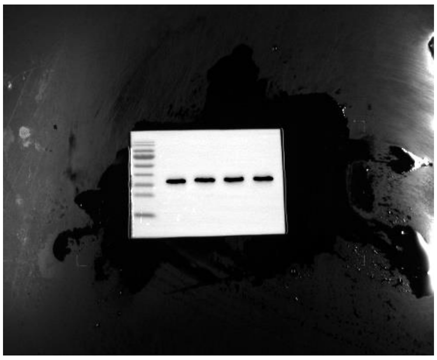

Figure 1F

Bcl-2 (26 kDa)

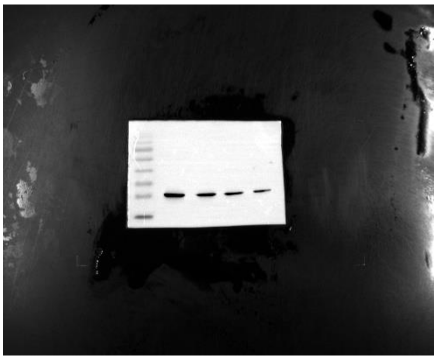

Caspase-3 (17 kDa)

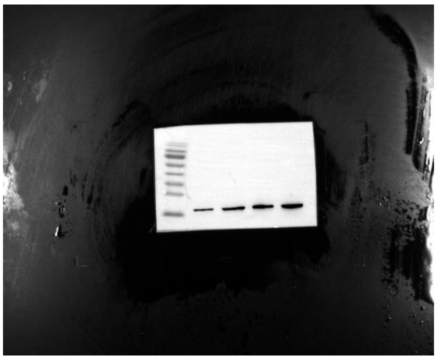

Bax (21 kDa)

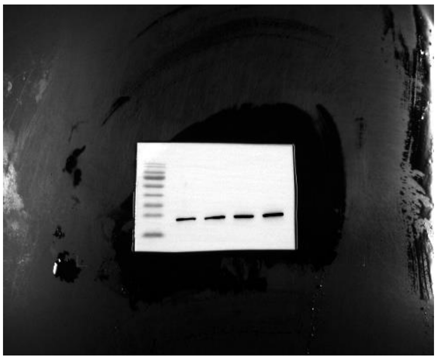

GAPDH (37 kDa)

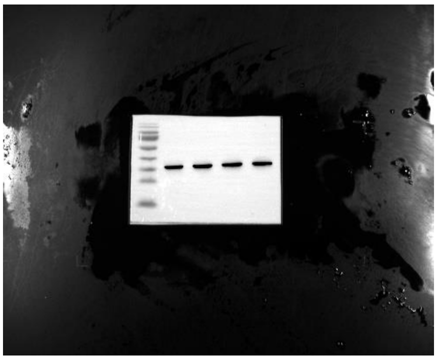

Figure 2A

Caspase 1 (45 kDa)

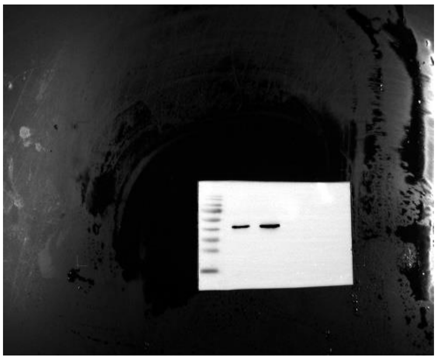

Gasdermin D (53 kDa)

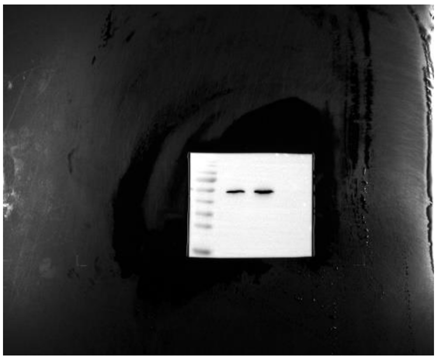

IL-1β (31 kDa)

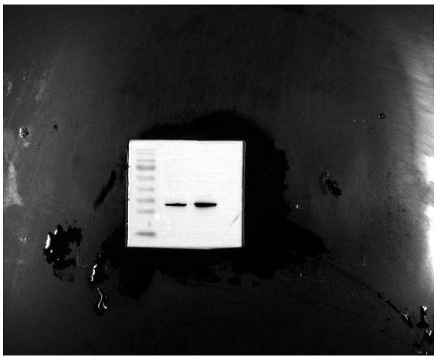

GAPDH (37 kDa)

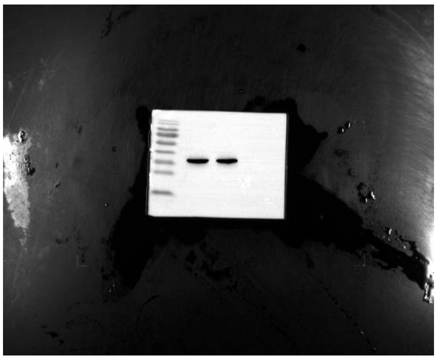

Figure 2B

IL-4 (17 kDa)

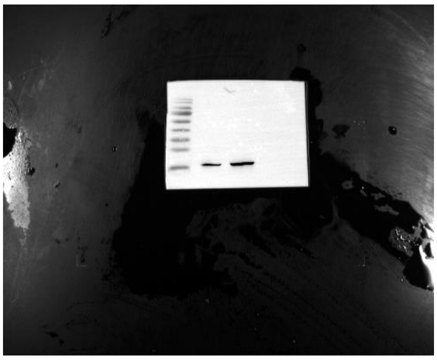

IL-6 (23 kDa)

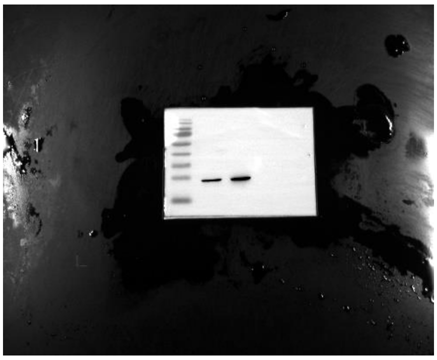

TNF-α (25 kDa)

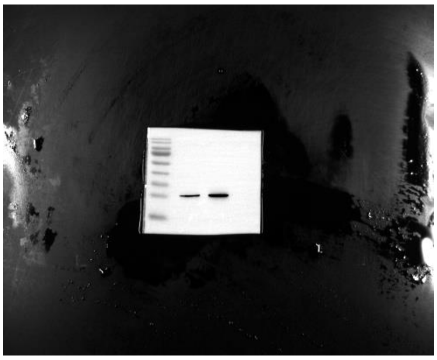

GAPDH (37 kDa)

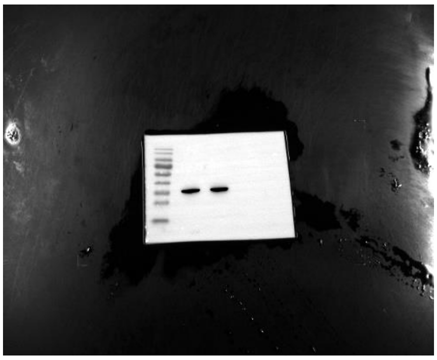

Figure 2C

Caspase 1 (45 kDa)

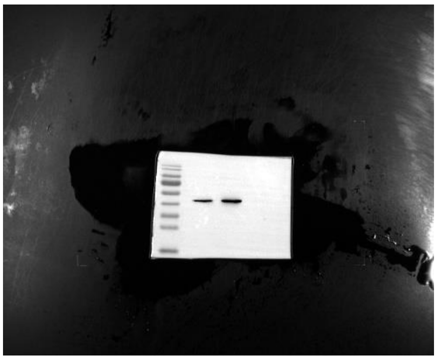

Gasdermin D (53 kDa)

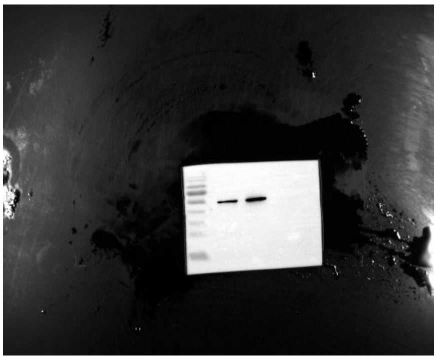

IL-1β (31 kDa)

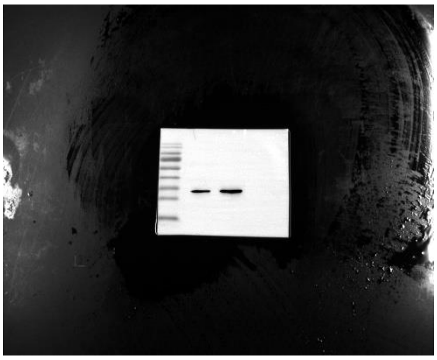

GAPDH (37 kDa)

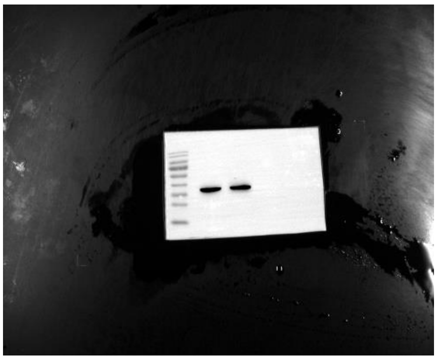

Figure 2D

IL-4 (17 kDa)

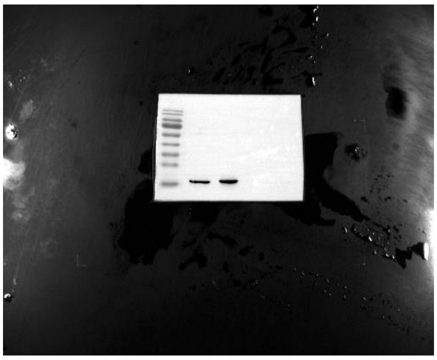

IL-6 (23 kDa)

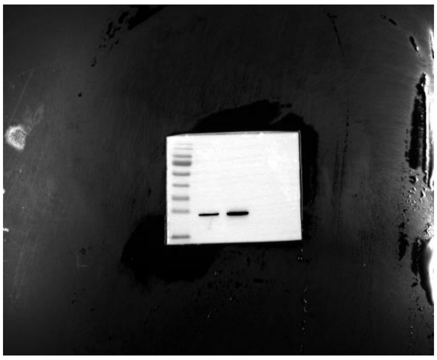

TNF-α (25 kDa)

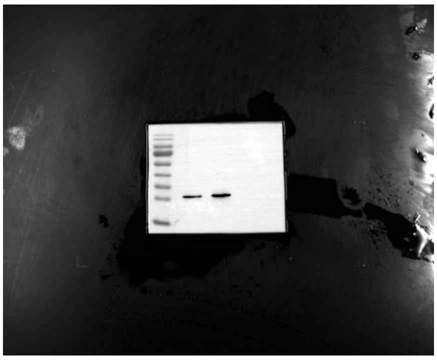

GAPDH (37 kDa)

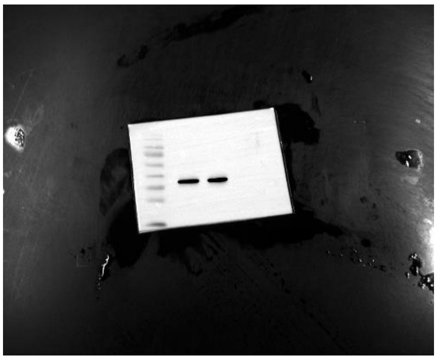

Figure 2F

α-SMA (42 kDa)

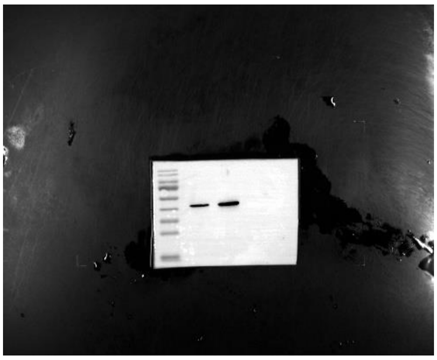

Col I (130 kDa)

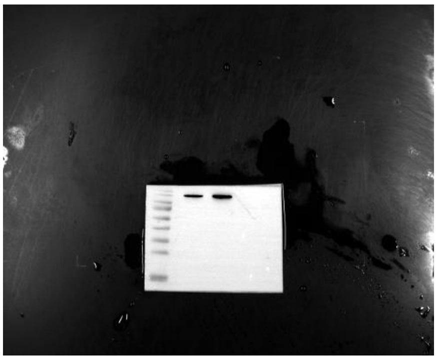

GAPDH (37 kDa)

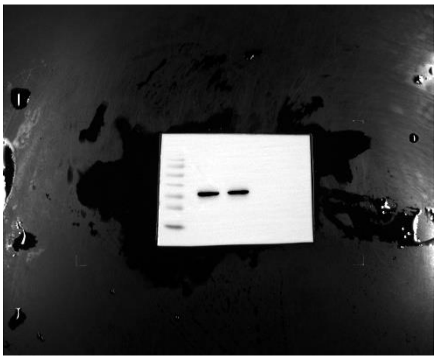

Figure 3A

Caspase 1 (45 kDa)

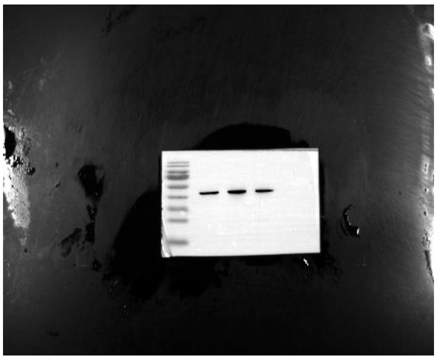

Gasdermin D (53 kDa)

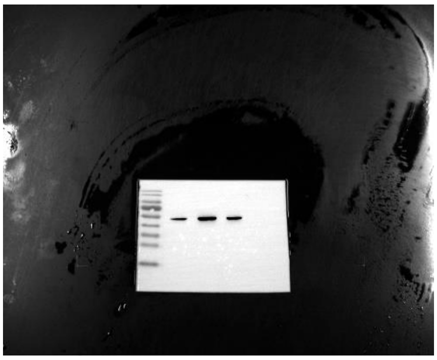

IL-1β (31 kDa)

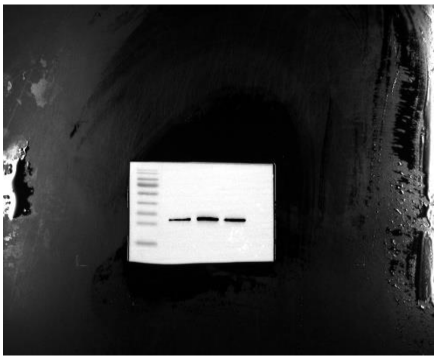

GAPDH (37 kDa)

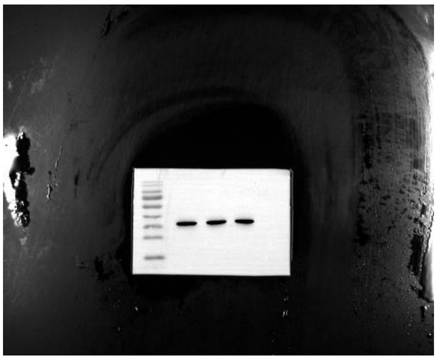

Figure 3B

Caspase 1 (45 kDa)

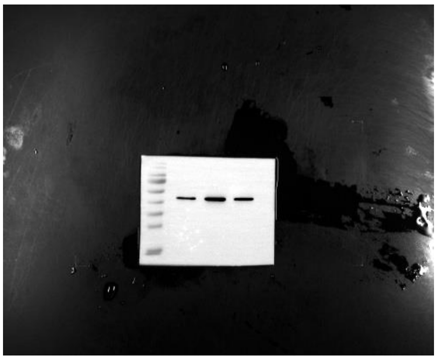

Gasdermin D (53 kDa)

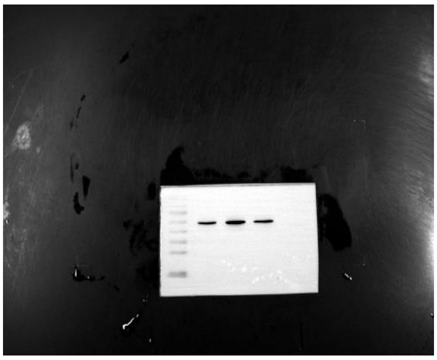

IL-1β (31 kDa)

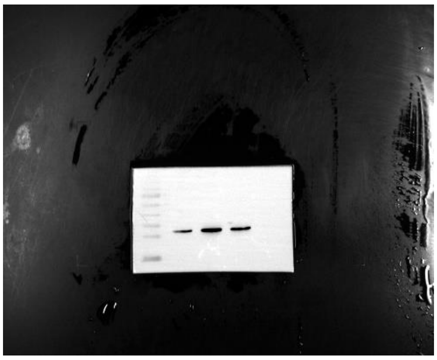

GAPDH (37 kDa)

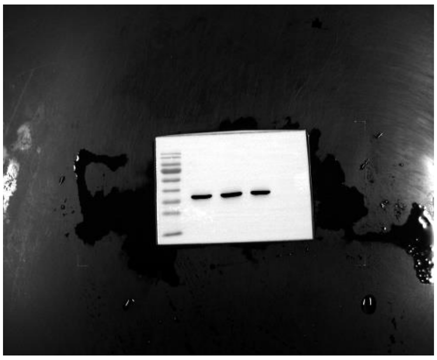

Figure 3C

IL-4 (17 kDa)

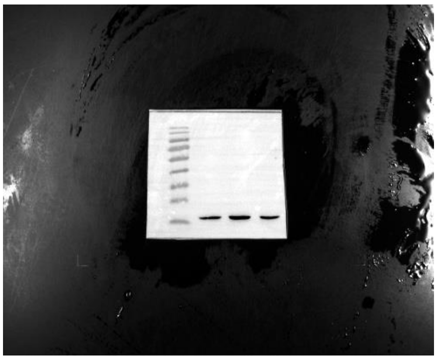

IL-6 (23 kDa)

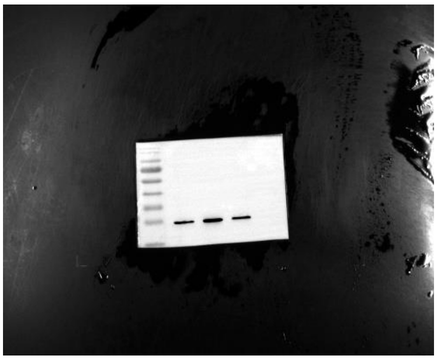

TNF-α (25 kDa)

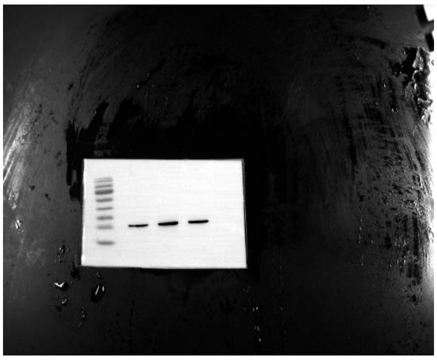

GAPDH (37 kDa)

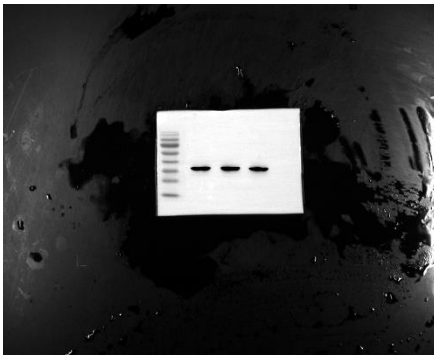

Figure 3D

IL-4 (17 kDa)

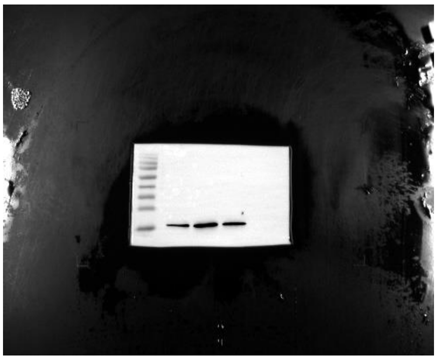

IL-6 (23 kDa)

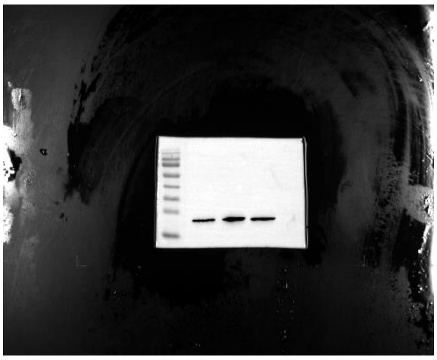

TNF-α (25 kDa)

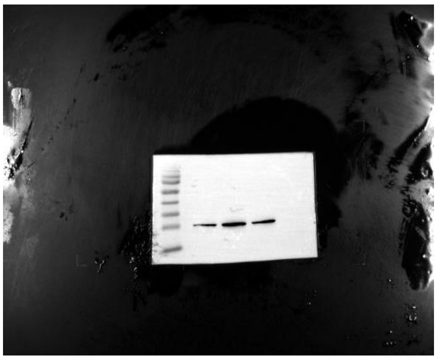

GAPDH (37 kDa)

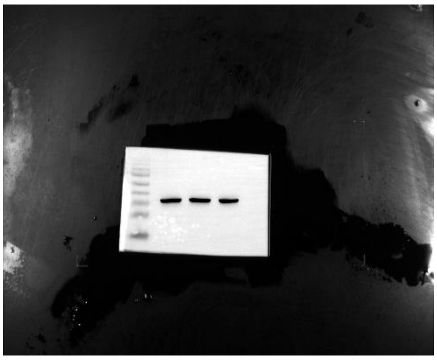

Figure 3J

α-SMA(42 kDa)

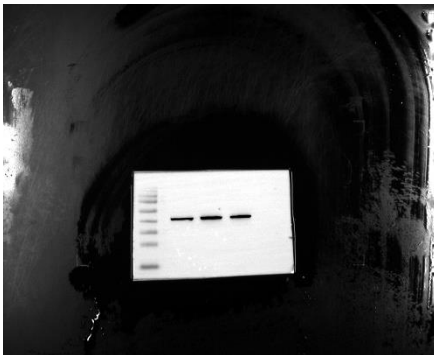

Col I (130 kDa)

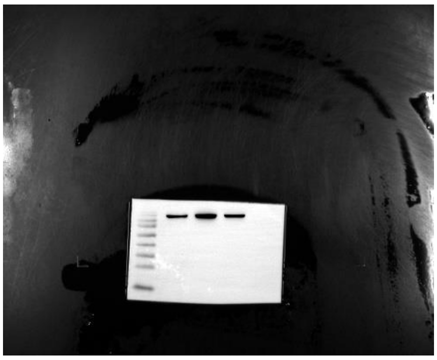

GAPDH (37 kDa)

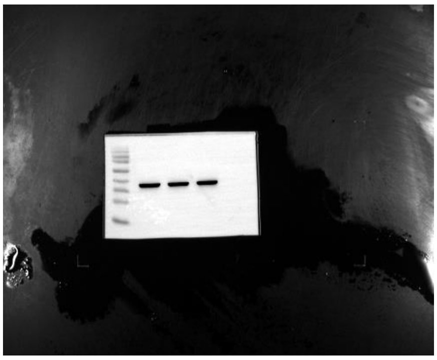

Figure 3K

Caspase 1 (45 kDa)

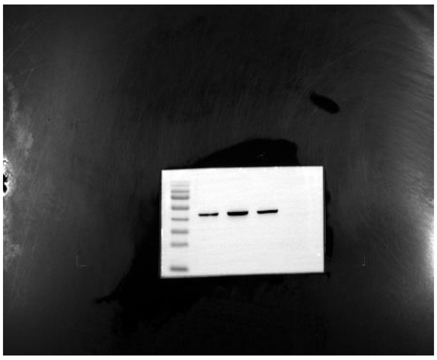

Gasdermin D (53 kDa)

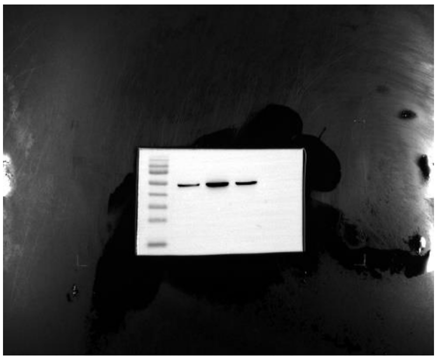

GAPDH (37 kDa)

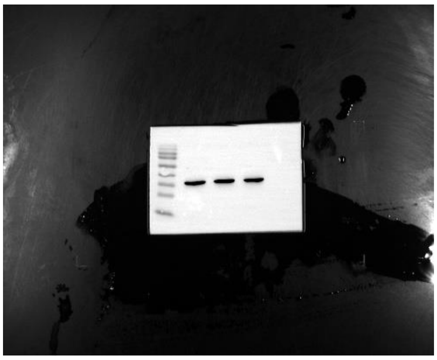

Figure 4C

MYD88 (33 kDa)

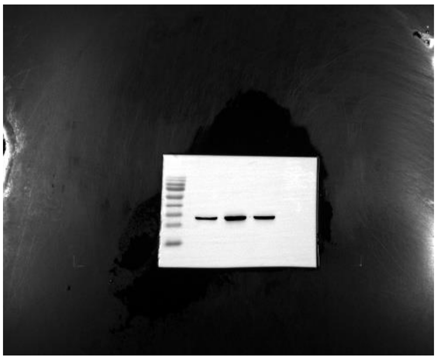

GAPDH (37 kDa)

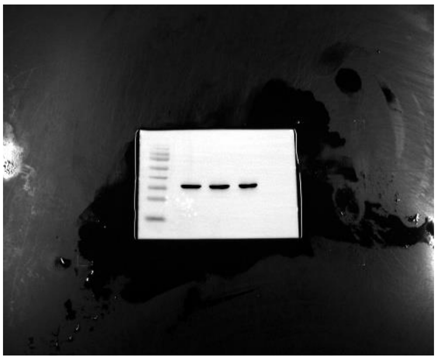

Figure 4D

MYD88 (33 kDa)

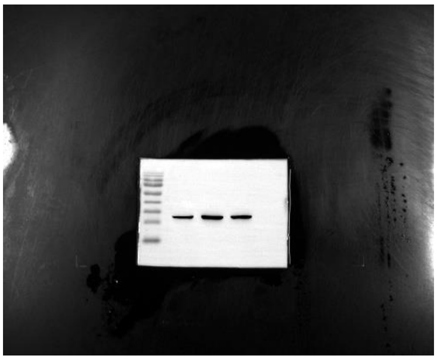

GAPDH (37 kDa)

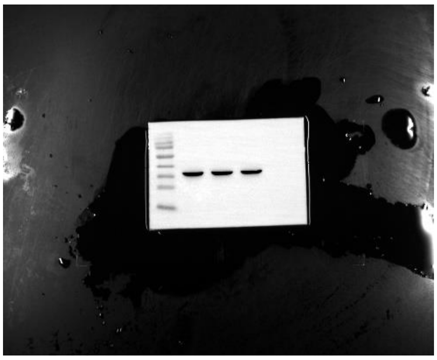

Figure 4G

MYD88 (33 kDa)

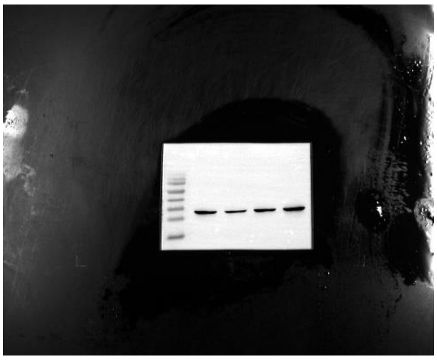

GAPDH (37 kDa)

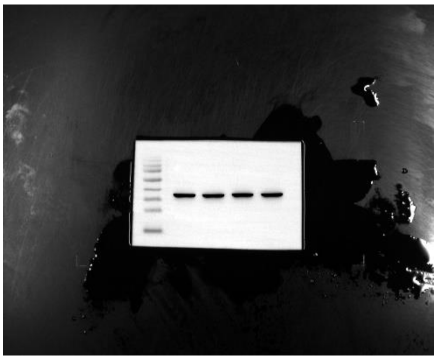

Figure 4H

IL-4 (17 kDa)

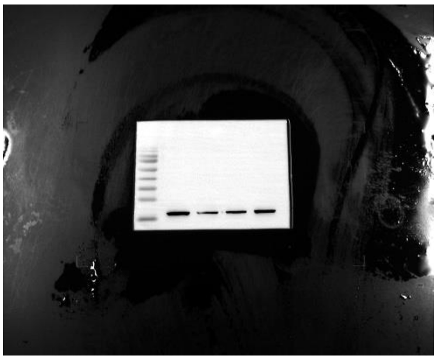

IL-6 (23 kDa)

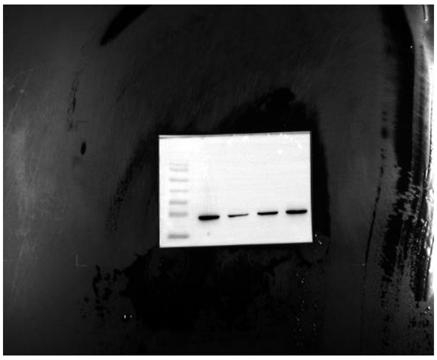

TNF-α (25 kDa)

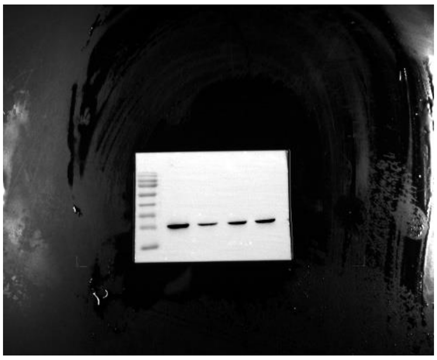

GAPDH (37 kDa)

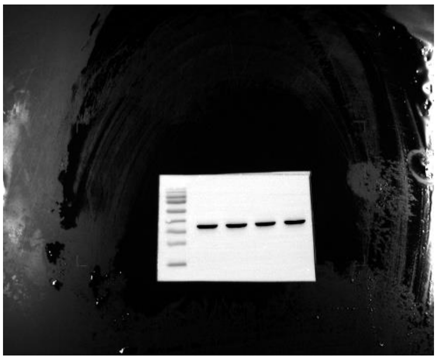

Figure 4I

IL-4 (17 kDa)

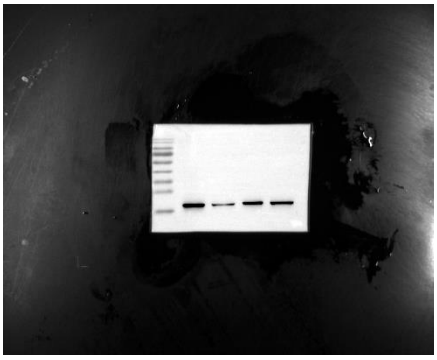

IL-6 (23 kDa)

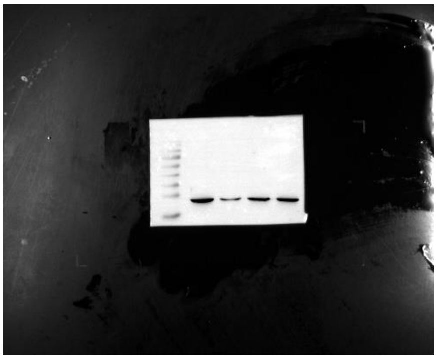

TNF-α (25 kDa)

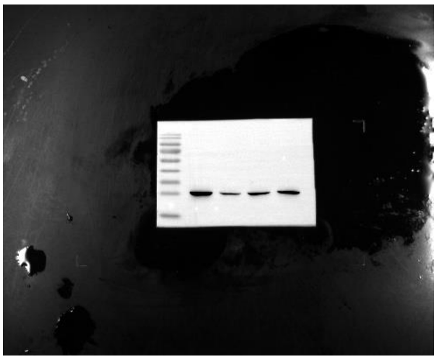

GAPDH (37 kDa)

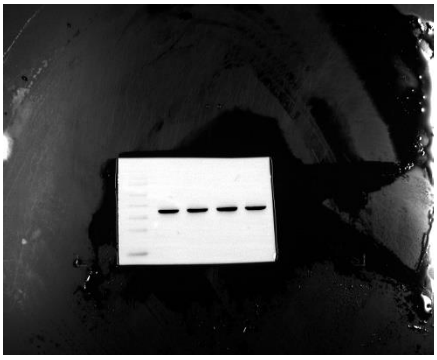

Figure 4J

Caspase 1 (45 kDa)

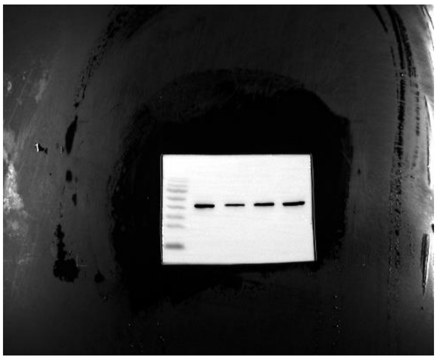

Gasdermin D (53 kDa)

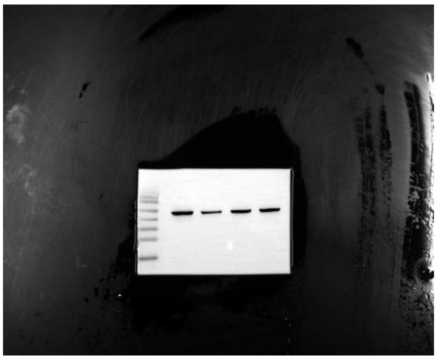

IL-1β (31 kDa)

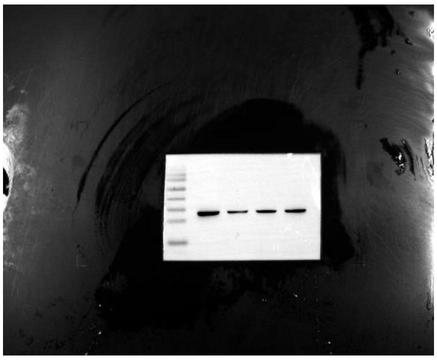

GAPDH (37 kDa)

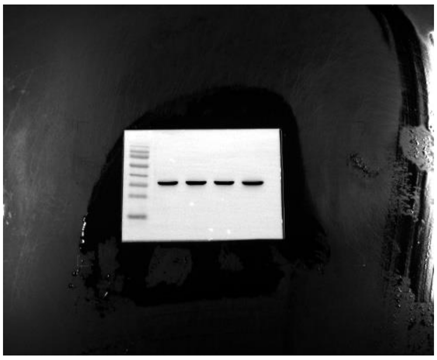

Figure 4K

Caspase 1 (45 kDa)

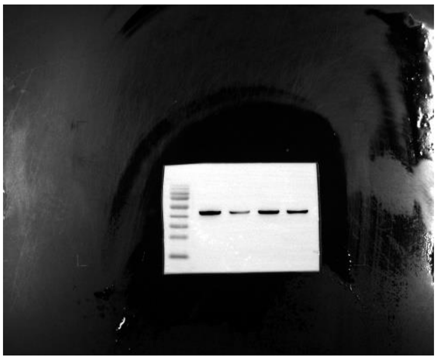

Gasdermin D (53 kDa)

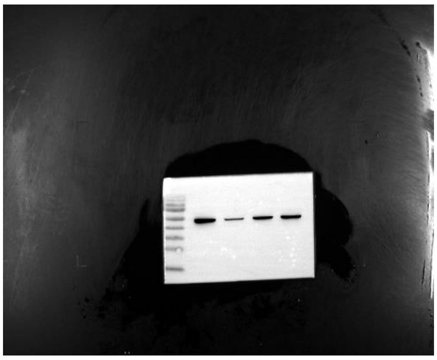

IL-1β (31 kDa)

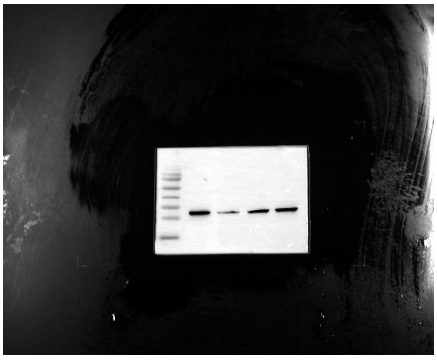

GAPDH (37 kDa)

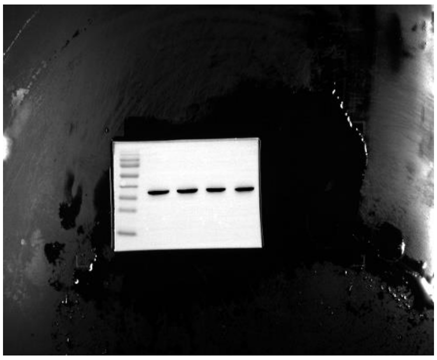

Figure 5F

α-SMA(42 kDa)

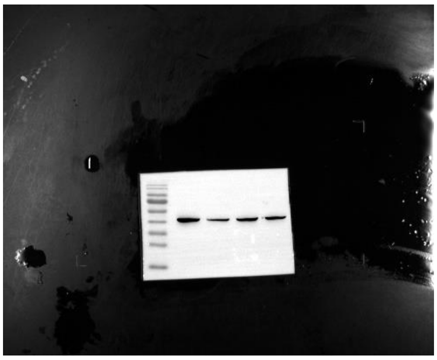

Col I (130 kDa)

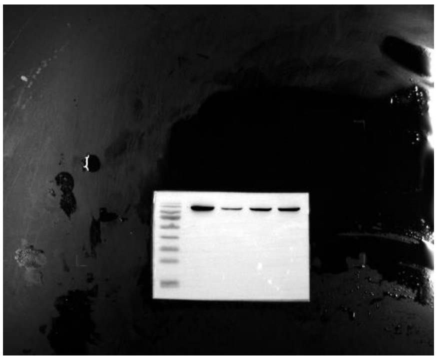

GAPDH (37 kDa)

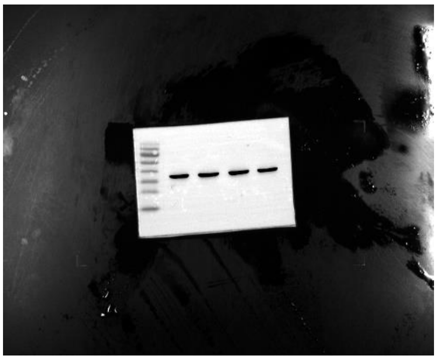

Supplement: S1 Raw images — (PDF) [file pone.0305409.s001.pdf]
